# High Spatial Resolution Cardiovascular Magnetic Resonance at 7.0 Tesla in Patients with Hypertrophic Cardiomyopathy – First Experiences: Lesson Learned from 7.0 Tesla

**DOI:** 10.1371/journal.pone.0148066

**Published:** 2016-02-10

**Authors:** Marcel Prothmann, Florian von Knobelsdorff-Brenkenhoff, Agnieszka Töpper, Matthias A. Dieringer, Etham Shahid, Andreas Graessl, Jan Rieger, Darius Lysiak, C. Thalhammer, Till Huelnhagen, Peter Kellman, Thoralf Niendorf, Jeanette Schulz-Menger

**Affiliations:** 1 Berlin Ultrahigh Field Facility (B.U.F.F.), Max-Delbrueck Center for Molecular Medicine, Berlin, Germany; 2 Working Group on Cardiovascular Magnetic Resonance, Experimental and Clinical Research Center, a joint cooperation between the Charité Medical Faculty of the Humboldt University of Berlin and the Max-Delbrueck Center for Molecular Medicine, and HELIOS Klinikum Berlin Buch, Department of Cardiology and Nephrology, Berlin, Germany; 3 MRI.TOOLS GmbH, Berlin, Germany; 4 National Institutes of Health / NHLBI, Bethesda, Maryland, United States of America; 5 DZHK (German Centre for Cardiovascular Research), partner site Berlin, Germany; University of Miami Miller School of Medicine, UNITED STATES

## Abstract

**Background:**

Cardiovascular Magnetic Resonance (CMR) provides valuable information in patients with hypertrophic cardiomyopathy (HCM) based on myocardial tissue differentiation and the detection of small morphological details. CMR at 7.0T improves spatial resolution versus today’s clinical protocols. This capability is as yet untapped in HCM patients. We aimed to examine the feasibility of CMR at 7.0T in HCM patients and to demonstrate its capability for the visualization of subtle morphological details.

**Methods:**

We screened 131 patients with HCM. 13 patients (9 males, 56 ±31 years) and 13 healthy age- and gender-matched subjects (9 males, 55 ±31years) underwent CMR at 7.0T and 3.0T (Siemens, Erlangen, Germany). For the assessment of cardiac function and morphology, 2D CINE imaging was performed (voxel size at 7.0T: (1.4x1.4x2.5) mm^3^ and (1.4x1.4x4.0) mm^3^; at 3.0T: (1.8x1.8x6.0) mm^3^). Late gadolinium enhancement (LGE) was performed at 3.0T for detection of fibrosis.

**Results:**

All scans were successful and evaluable. At 3.0T, quantification of the left ventricle (LV) showed similar results in short axis view vs. the biplane approach (LVEDV, LVESV, LVMASS, LVEF) (p = 0.286; p = 0.534; p = 0.155; p = 0.131). The LV-parameters obtained at 7.0T where in accordance with the 3.0T data (p_LVEDV_ = 0.110; p_LVESV_ = 0.091; p_LVMASS_ = 0.131; p_LVEF_ = 0.182). LGE was detectable in 12/13 (92%) of the HCM patients. High spatial resolution CINE imaging at 7.0T revealed hyperintense regions, identifying myocardial crypts in 7/13 (54%) of the HCM patients. All crypts were located in the LGE-positive regions. The crypts were not detectable at 3.0T using a clinical protocol.

**Conclusions:**

CMR at 7.0T is feasible in patients with HCM. High spatial resolution gradient echo 2D CINE imaging at 7.0T allowed the detection of subtle morphological details in regions of extended hypertrophy and LGE.

## Introduction

Cardiovascular magnetic resonance (CMR) is known to offer additional morphologic information in hypertrophic cardiomyopathy (HCM). Accurate phenotyping is essential for the diagnosis and risk stratification of HCM [1]. Echocardiography is currently the most important basic imaging modality in the diagnostic work-up of patients and relatives [2]. CMR is able to provide information beyond myocardial function based on CINE-imaging by detecting fibrosis based on Late Gadolinium Enhancement (LGE) imaging [3]. Fibrosis imaging plays an important role in risk stratification of HCM. It is accepted as a “modifier” in the HCM guidelines [1]. In a comprehensive evaluation of HCM, CMR-based myocardial tissue differentiation with assessment of perfusion and fibrosis provides important information [4,5]. An ongoing multi-center international trial (Hypertrophic Cardiomyopathie Registry-HCMR) includes fibrosis imaging based on LGE and T_1_-mapping and will help to define the role of CMR in risk stratification [6].

Furthermore, CMR allows the identification of focal hypertrophy in atypical regions. In particular the apical and the anterolateral region may be underestimated with echocardiography [7]. In recent years, small morphological features such as myocardial crypts or clefts have come to awareness. They have been described increasingly in different genotypes, but are not specific for HCM [8,9]. The detection of small myocardial structures goes along with the improvement of clinically available imaging technology, mainly with an increased spatial resolution.

Currently, experimental MRI at 7.0 Tesla (T) is under evaluation in a human setting, mainly covering neuroscience. Neurovascular ultrahigh field (UHF)-MR has been successfully performed in different clinical entities. Based on the increased spatial resolution MRI at 7.0T was superior to 3.0T [10]. Early applications of UHF-CMR manifest the enhancements in spatial resolution, but were limited to healthy volunteer studies [11–15].

The aim of our study was to prove the feasibility of CMR at 7.0T in HCM and investigate its capability for the detection of subtle morphological changes in comparison to standardized clinical protocols.

## Methods

The ethics committee (Charite Campus Mitte EA1/54/09) approved the study and all participants provided written informed consent prior to the study. (Ethics committee: Ethicausschuss 1 am Campus Charite Mitte head: Prof. Dr. R Uebelhack Charitéplatz 1, 10117 Berlin phone: +4030450–517222 Ethics approval number: EA1/054/09, Renewal number: NI 532/6-2)

### Study population

We prospectively screened patients with HCM. As a reference group healthy volunteers were identified for eligibility for 7.0T.

### Exclusion criteria

Usual MR-exclusion criteria such as claustrophobia and implanted devices were applied. In particular at 7.0T, all metallic implants and tattoos led to an exclusion. Furthermore, all patients with any evidence of other cardiovascular diseases, severe arrhythmias and renal failure based on the estimation of glomerular filtration reserve < 60ml/min were excluded.

### Patients

The diagnosis of HCM was based on clinical parameters including echocardiography following the guidelines [1].

### Healthy volunteers

Healthy volunteer was defined based on clinical investigation and a negative history of any diseases. There were no ECG-abnormalities and cardiac function was normal.

### CMR-protocol

#### CMR at 7.0 Tesla

A whole body 7.0T MR-system (Magnetom, Siemens Healthcare, Erlangen, Germany, equipped with a gradient system providing a maximum gradient strength of 38 mT/m and a maximum slew rate of 170 mT/m/ms (Siemens Healthcare, Erlangen, Germany) were used. For signal reception and transmission, a 16-channel radio-frequency (RF) transceiver array tailored for CMR at 7.0T was employed [14]. Prior to the study, the RF coil underwent thorough safety assessment in line with the technical standards given by IEC 60601-2-33:2010 Ed.3 and IEC 60601–1:2005 Ed.3 [16]. The safety assessment, the implemented safety measures, the technical documentation and the risk management file for the coil were evaluated and duly approved for implementation in clinical studies following conformity declaration provided by a notified body.

The basic scan protocol was described recently [11,12]. In brief, 2D CINE FLASH images were acquired using a high resolution fast gradient echo (FGRE) technique in end-expiratory breath-holds. Imaging parameters were: echo time (TE) = 2.7 ms, repetition time (TR) = 5.5 ms, nominal flip angle (FA) = 32°, field of view (FOV) typically (360x360) mm^2^, FOV phase = 73%, acquisition matrix size = 256×186, bandwidth (BW) = 445 Hz/pixel, 30 phases per heart cycle, parallel imaging using two-fold acceleration and GRAPPA reconstruction (R = 2). We acquired three long-axis views of the left ventricle ((slice thickness (slth) = 4.0mm)) corresponding to the standard procedure in clinical routine. Additionally, three short axes views (slth 4.0mm and 2.5mm) were acquired in the LGE-positive region as identified at 3.0T. Specific slices were acquired to enhance regions with noticeable structure as identified in clinical scans at 3.0T. Cardiac gating was performed with acoustic cardiac triggering (easyACT, MRI.TOOLS GmbH, Berlin, Germany) [17] or pulse oximetry.

#### CMR at 3.0 Tesla

A 3.0T MR system (Magnetom Verio, Siemens Healthcare, Erlangen, Germany) was used. For signal transmission, a whole body RF coil was applied. For signal reception a 32-channel RF coil dedicated for CMR was employed.

According to the established clinical protocol, 2D steady-state free precession (SSFP) CINE imaging was applied for cardiac chamber quantification. We acquired four-, two—and three chamber views and a stack of short axis views covering the whole left ventricle without a gap (slth: 6.0mm, TR: 3.1ms, TE: 1.3ms FA: 45°, FOV: (340 x 276) mm^2^, matrix: 192x156, BW: 704Hz/px, 30 phases per heart cycle, GRAPPA reconstruction, acceleration factor 2 [18]. LGE images were acquired 10 to 15 minutes after application of gadobutrol (0.2mmol/kg body weight) using fast low angle shot (FLASH) inversion recovery gradient echo to detect fibrosis. Imaging parameters were: TR = 10.5ms, TE = 5.4ms, FA = 30°, FOV (350 x 262) mm^2^, matrix 256 x162, slth 6.0mm, BW 140Hz/px, GRAPPA acceleration factor 2. Cardiac gating was performed using ECG.

#### CMR at 1.5 Tesla

A subgroup of our study population (n = 2) underwent an additional scan at 1.5T (MAGNETOM Avanto, Siemens Healthcare, Erlangen, Germany) using a 12-channel RF body array coil for signal reception. 2D CINE images were acquired using SSFP following our routine protocol. Imaging parameters were: TE = 2.7 ms, TR = 5.5 ms, FA = 80°, FOV typically (340x340) mm^2^, matrix 192×156, slth 7.0mm, 30 phases per heart cycle, parallel imaging with two-fold acceleration and GRAPPA reconstruction. Pre-contrast multi–echo fat–water–separated imaging was applied as following: bandwidth = 977 Hz/pixel, matrix = 256×126, TR = 11.2 ms, TE = 1.64, 4.17, 6.7, and 9.23 ms, flip angle = 20–25° [19].

### Image analysis

#### Quantitative analysis

LV morphology was quantified using CVI^42^ version 4.15 (Circle Cardiovascular Imaging, Calgary, Canada). LV myocardium was delineated by semi-automatically contouring the endocardial and epicardial borders. For LV quantification, both biplanar (3.0T, 7.0T) and short axis data were analyzed (3.0T). The papillary muscles were excluded from LV-mass and counted as blood in the biplanar approach reflecting the area-length method as published, whereas the LV quantification based on the short axis stack regarded the papillary muscles as myocardium [20].

#### Qualitative analysis

Image quality of the CINE images at 7.0T was analyzed qualitatively. Artifacts and anatomical particularities were assessed as published recently [12]. Quality score were 0 = non-diagnostic, 1 = good, and 2 = excellent. The artifact-score was as following: 2 = major artifacts, 1 = mild artifacts, 0 = no artifacts. Two experienced observers (FVK > 10000 CMR scans, MP > 1500 CMR scans) evaluated a mid-ventricular short axis view with different slice thicknesses (4.0 and 2.5mm) and a three chamber view (4mm). Visual assessment of pericardial effusion was based on CINE imaging evaluating the pericardial bright signal following clinical criteria. Wall motion abnormalities were visually scored following established criteria (normo-, hypo-, a- and dyskinesia).

All images were systematically screened for subtle morphological abnormalities such as the myocardial structure itself and papillary muscles. The visual evaluation was slice based and an evaluation of the perpendicular slices using a cross-reference tool was allowed. The definition of myocardial crypts was based on previous studies [9].

## Statistics

Results are presented as mean ± standard deviation. The Wilcoxon matched pairs test was used to compare the results in HCM patients. The Mann-Whitney test was used to compare the results between healthy volunteers and patients. Statistical significance was accepted as p<0.05. Statistical analyses were performed using SPSS version 20.0 (IBM, Armonk) and Prism version 5.0 (graphpad, San Diego).

## Results

### Study population

We screened 131 patients with HCM between 2011 and 2014. Main reasons for exclusion at 7.0 T were implants like cardiac devices (n = 16), significant cardiac-morbidity (n = 32), other co-morbidities (35) and arrhythmias (n = 7). Because of the rigorous exclusion criteria we had to exclude patients with dental implants (n = 16), tattoos (7). Some patients refused to participate in the 7.0 T study (n = 18).

**Fig 1:**
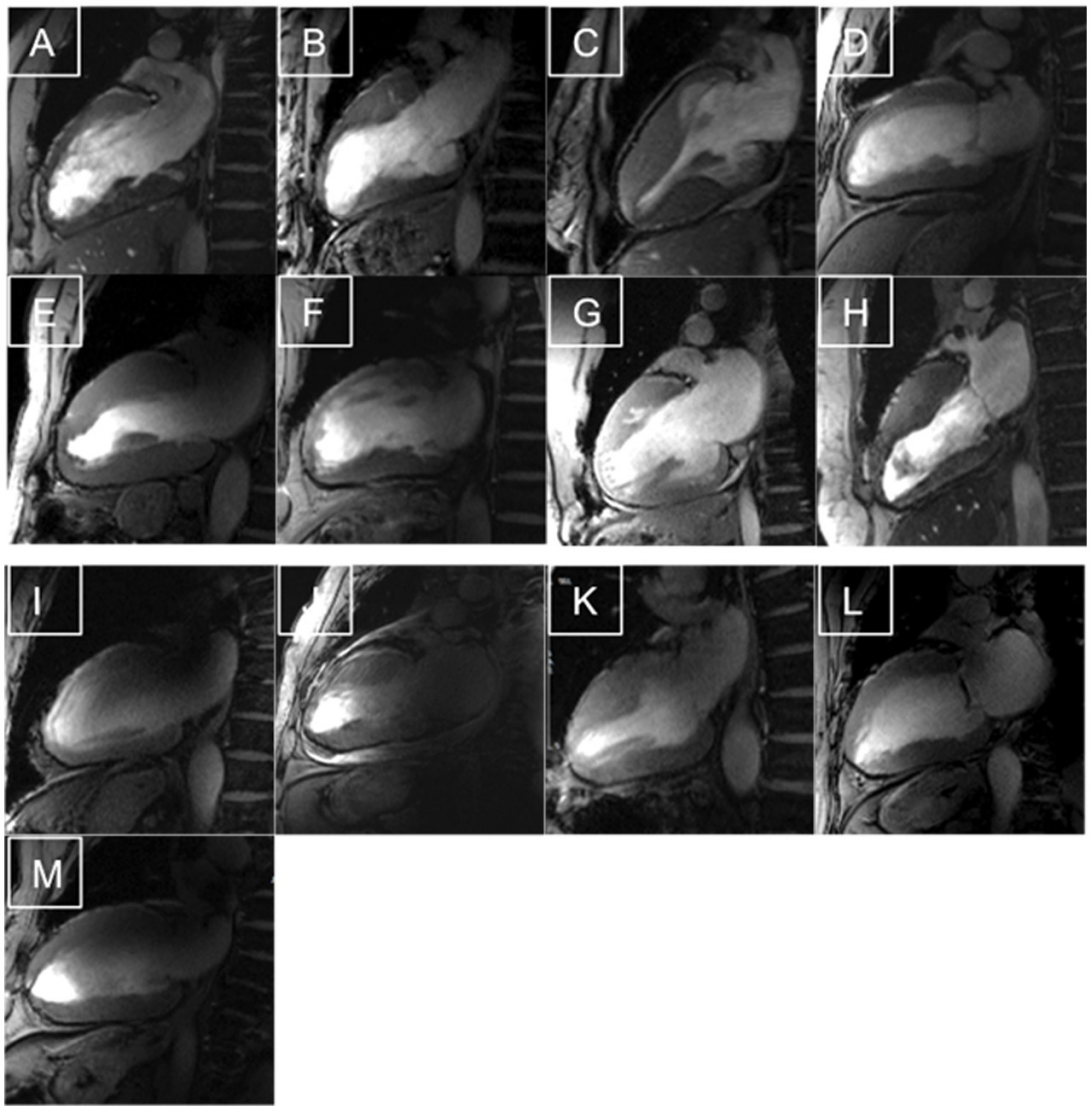
Feasibility of CMR in HCM patients at 7.0T. High Resolution CINE images of each patient (slice thickness 2.5 mm) All images were evaluable as shown by these two-chamber views, but the quality scoring revealed differences. A-H) Examples with a good images quality and mild artifacts. I-M) Images with different types of artifacts

26 participants (13 HCM patients) were included and successfully scanned at 7.0T and at clinical scanners (mean time interval 29 days) (Fig 1). Mean scan time at 7.0T assessing the LV-morphology was 22±13 minutes. All scans could be performed without any complication. Only temporary dizziness, temperature sensations and metallic taste were reported in 6 cases at 7.0T.

### HCM-Patients

We examined 9 males (mean age: 56 ±31) and 4 females (mean age: 54 ±12) at 7.0T (Table 1). No relevant symptoms were reported during or after the 7.0T-scan.

**Table 1:** Clinical characteristics and left ventricular assessment for healthy volunteers and HCM patients at 7.0 T. BMI—Body Mass Index, LVEDV—Left Ventricular End-Diastolic Volume, LVEF—Left Ventricular Ejection Fraction, LVESV—Left Ventricular End-Systolic Volume, PE—Pericardial Effusion, WMA—Wall Motion Abnormalities.

	patients with HCM	healthy volunteers
**gender**	13 (9 male)	13 (9 male)
**age (years)**	56 (25–71)	55 (24–71)
**BMI (kg/m**^**2**^**)**	27 (22–36)	24 (19–29)
**Dental wires(n)**	11	4
**WMA(n)**	1	-
**PE(n)**	1	-
**Crypts(n)**		-
**- absolute**	7	
**- average/patient**	1	
**- maximum/patient**	3	
**LGE(n)**	12	-
**LVmass (g)**	174.9 (112.8–273.5)	100.3 (75.2–134.5)
**LVEDV (ml)**	136.7 (68.5–231.2)	127.2 (94.9–186.8)
**LVEF (%)**	59.9 (50.2–76.0)	58.5 (49.8–71.6)
**LVESV (ml)**	51.2 (26.3–71.3)	55.4 (35.8–75.8)

All patients underwent also CMR at clinical field strengths. Eleven patients were investigated at 3.0T, two patients refused the examination. In these cases we evaluated the clinical scan at 1.5 T. The clinical CMR protocol at 1.5T assessing LV function and fibrosis was similar to the 3.0T-protocol.

### Healthy Volunteers

Nine male and four female healthy volunteers completed the scan at 7.0T. They were age- and gender-matched to HCM (Table 1). No relevant symptoms during or after the 7.0T-scan were reported.

### Qualitative image analysis

The image quality score of 7.0T 2D CINE FGRE data reflected a good quality. The quality was scored as”good” with a mean of 1.1±0.3 by observer 1 and 1.3±0.2 by observer 2 (FvK,MP). Artifacts were identified in six patients, but they did not influence the evaluation of the cardiac function. The mean artifact score was classified as mild reflected by a score of 1.2±0.2 given by observer 1 and 1.4±0.3 by observer 2.

**Fig 2:**
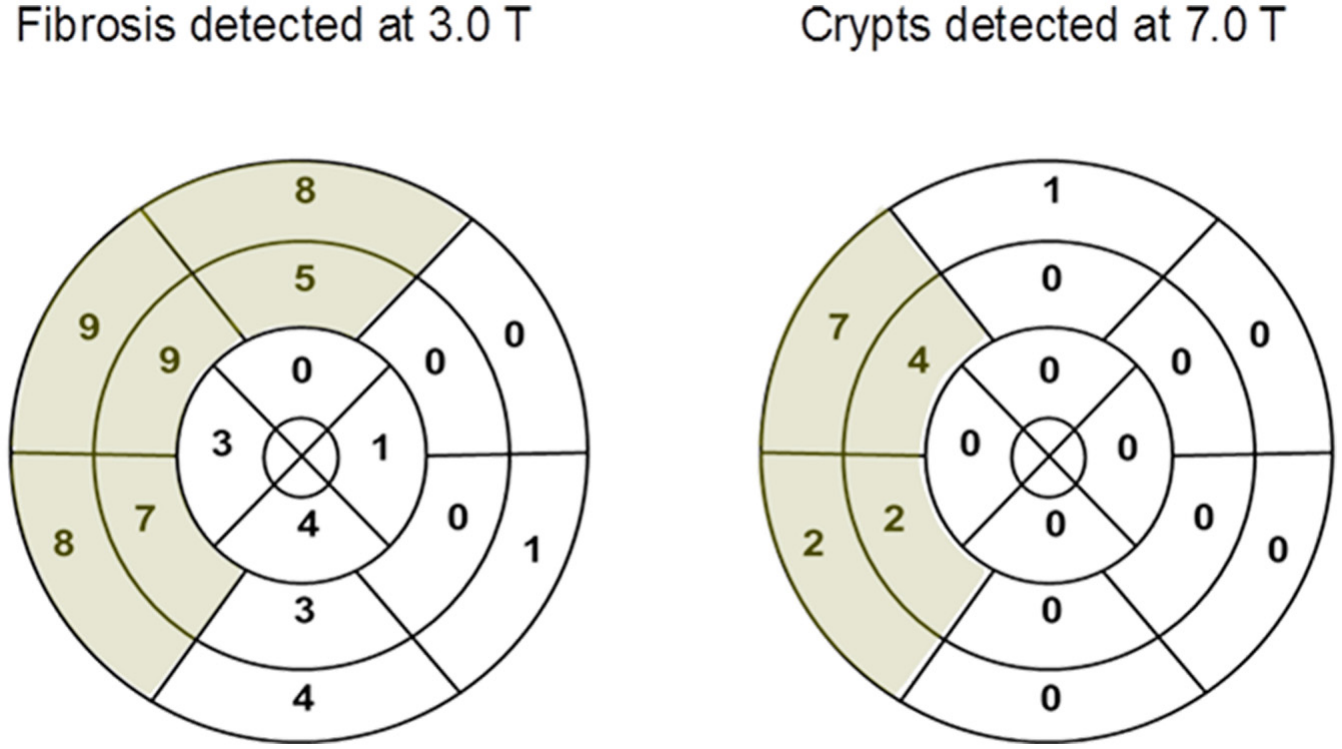
Distribution and prevalence of fibrosis and crypts. Myocardial crypts were only located in the regions with fibrosis as identified by LGE at 3.0T

**Fig 3:**
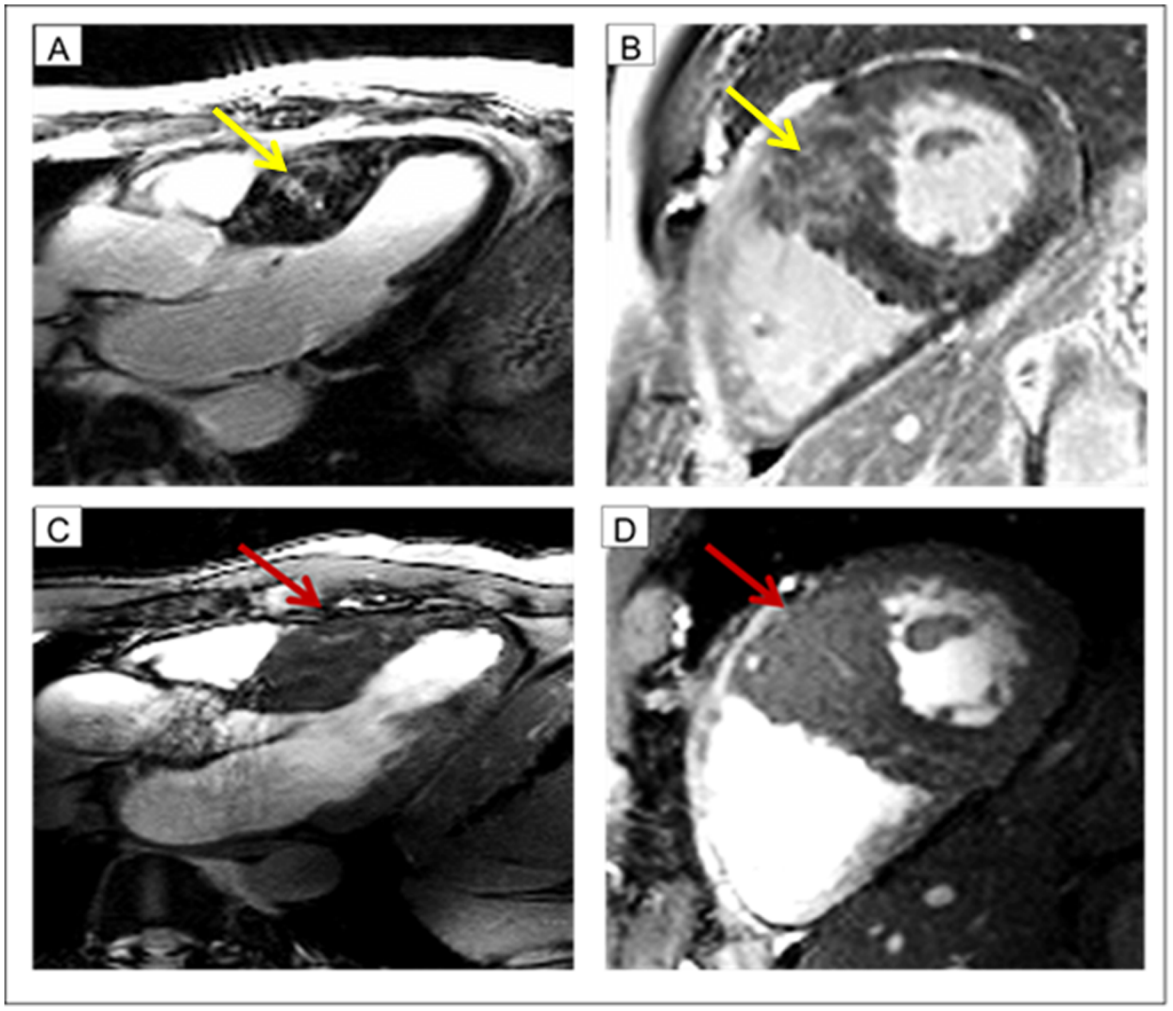
Case example: Patient with myocardial crypts. In the top row fibrosis imaging (LGE at 3.0T) is shown. The yellow arrow indicates the fibrosis (A long axis view B short axis view). In the bottom row cine imaging at 7.0T is shown The red arrow indicates the myocardial crypts (A long axis view B short axis view). Remarkable, fibrosis and crypts have a certain overlap. One may assume, that the bright signal at 3.0T might be also induced by blood within the crypts.

**Fig 4:**
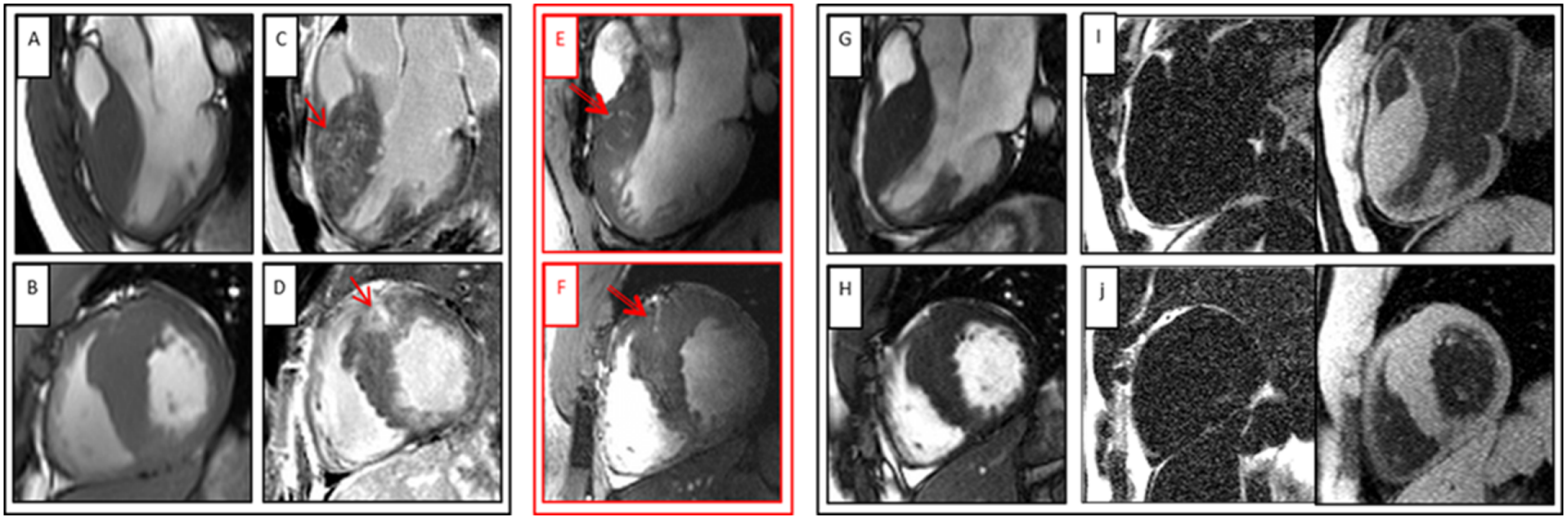
HCM patient with myocardial crypts in the anteroseptal region. Upper row: 3-chamber view with different techniques, Bottom row: short axis view using the same techniques, A and B CINE images at 3.0T, C and D Late Gadolinium Enhancement at 3.0T, E and F CINE images at 7.0T, G and H CINE images at 1.5 T CMR, I and J Fat-Water images, Single arrows indicates LGE at 3.0T, Double arrow displays myocardial crypt at 7.0T.

The visual evaluation of the 7.0T CINE images acquired with a slice thickness of 2.5mm and 4.0mm and an in-plane spatial resolution of (1.4x1.4) mm^2^ revealed unexpected results of the myocardial structure in the areas of LGE depicted at 3.0T (distribution of LGE see Fig 2). At 7.0T, we detected small hyperintense regions in the myocardium mainly in the hypertrophied anteroseptal region. Most of them had access to the blood of the left ventricle. The visibility of the hyperintense regions was related to the cardiac phase. The structures were best detectable during diastole. The findings could be identified as myocardial crypts (Fig 3 and S1 File). To exclude the theoretical possibility of fatty infiltration as the cause of the hyperintense signal, we re-scanned two patients at 1.5T applying multi–echo fat–water–separated imaging. No fat was detectable in the respective regions (Fig 4).

Myocardial crypts were observed in 7/13 (54%) of HCM patients at 7.0T. The agreement of two experienced (MP and FvK) readers was 92%. Prospective and retrospective analysis of the corresponding CINE images at the clinical field strengths did not allow the detection of intramyocardial hyperintense structures. No myocardial crypts were detected in healthy volunteers.

### Quantitative analysis of the left ventricle

**Fig 5:**
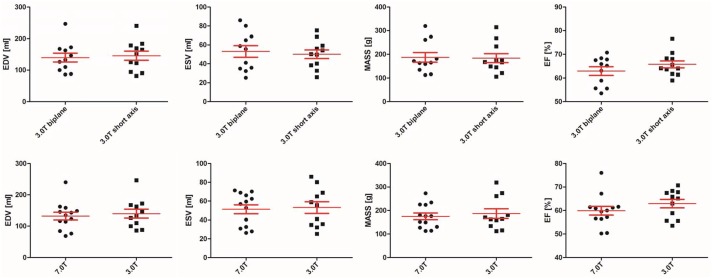
Comparison of left ventricular function between the different field strengths. The comparison of left ventricular function revealed no ignificant differences between the field strengths, Top: Left ventricular function at 3.0 T (biplanar versus short axis). Bottom: Left ventricular function at 7.0 T compared to 3.0 T (both biplanar).

There were no significant differences between short axis and long axis assessment at 3.0T: LVEDV (p = 0.286), LVESV (p = 0.534), LVEF (p = 0.131) and LV-Mass (p = 0.155). The long axis comparison between 3.0T and 7.0T also revealed no significant differences: LVEDV (p = 0.110), LVESV (p = 0.091), LVEF (p = 0.182) and LV-MASS (p = 0.131) (Table 2). Plots are shown in Fig 5.

**Table 2:** Left ventricular assessment of HCM patients at different field strengths. SAX = Short axis

	7.0T(biplanar)	3.0T(biplanar)	3.0T(sax)	p-value(3.0T biplanar vs. 3.0T sax)	p-value(7.0T biplanar vs 3.0T biplanar)
**LVEDV (ml)Mean**	**136.7**	**139.9**	**148.0**	**0.286**	**0.110**
**Min-Max**	**68.5–231.2**	**86.3–256.5**	**81.93–240.4**		
**LVESV (ml)Mean**	**51.2**	**53.0**	**50.0**	**0.534**	**0.091**
**Min-Max**	**26.3–71.3**	**25.3–85.9**	**26.0–75.3**		
**LVM (g)Mean**	**174.9**	**187.0**	**183.8**	**0.155**	**0.131**
**Min-Max**	**112.8–273.5**	**112.6–319.4**	**105.3–314.6**		
**LVEF (%)Mean**	**59.9**	**62.9**	**65.7**	**0.131**	**0.182**
**Min-Max**	**50.2–76.0**	**53.6–70.7**	**59.0–76.7**		

Quantitative analysis of LV function at 7.0T in healthy volunteers is shown in Table 1.

As expected, LV-mass differed significantly between healthy volunteers and HCM patients (p = 0.001). There were no significant differences in LVEDV (p = 0.541), LVESV (p = 0.316) and LVEF (p = 0.451) between field strength.

### Technical aspects

Magnetic field inhomogeneity did not influence the assessment of the cardiac structures. Cardiac gating was successful in all participants. In six volunteers we had to switch from acoustic gating to pulse oxymetry (four HCM patients and two healthy volunteers).

## Discussion

To the best of our knowledge, this is the first study showing the feasibility of CMR at 7.0T in a larger group of cardiac patients. Besides the successful implementation of 7.0T in HCM, we were able to identify unexpected subtle crypts in hypertrophied regions, which were not detectable at clinical field strengths in a routine setting [4,21]. The detection of various morphological changes may have impact on advanced non-invasive phenotyping of HCM.

### Quantitative assessment of left ventricle

The assessment of the left ventricular function is a basic requisite of a CMR scan. In our setting we could confirm that the LV-function obtained at 7.0T accords with the 3.0T data. Our findings are in line with previous results, that the assessment of cardiac function is reliable at 7.0T [11]. So far the assessment of cardiac function is not an obstacle to further application developments at ultrahigh field strengths.

### Qualitative assessment of left ventricle

Based on high-resolution CINE imaging, we have identified myocardial crypts at 7.0T. They were detectable in regions with hypertrophy and fibrotic changes as indicated by LGE. We detected the crypts in more than 50% of our HCM-patients applying high spatial resolution CINE imaging at 7.0T. Theoretically, the incidence of crypts could be even higher, as in our setting full coverage including different slice positions or high resolution 3D cine imaging was not applicable. The detected crypts were not observed at 3.0T or 1.5 T using standardized 2D CINE SSFP imaging. The findings may lead to new insights into the bright regions seen with LGE imaging (Fig 4). LGE itself is associated with an impaired clinical outcome based on heart failure and arrhythmias [4]. A further differentiation could enhance the prediction of different outcomes. The interpretation of hyperintense signal in non-contrast CINE imaging can be difficult, as it can be caused by fat or fluid [19]. We could exclude that the bright signal is caused by fat by using fat-water imaging at 1.5 T.

Crypts have already been described in HCM applying CMR [22,23]. Interestingly, they were found in HCM patients with LV-hypertrophy, but also in genotype-positive patients without LV-hypertrophy [9]. One group detected the crypts more often in non-hypertrophied regions and explained that by the remodeling process [9].

One could assume that the myocardial crypts are compressed by the hypertrophied myocardium. The currently used image spatial resolution at lower field strength is not good enough to depict them. During the time course of disease while developing heart failure, they could be detectable. The prevalence of myocardial crypts as detected at 7.0T seems to be as high as described in pre hypertrophy stages of HCM [8]. Systematic CMR based follow-up would offer the chance to depict this and to elucidate the underlying mechanisms. The description of subtle myocardial structural changes, such as crypts, may help to predict the disease development and the differentiation between risk of sudden cardiac death and development of heart failure.

Petryka et al reported a prevalence of crypts in HCM of 15.6% using both 1.5T and 3.0T. Crypts were mainly identified in the non-hypertrophied inferior wall [24]. In our study, the prevalence of crypts was 54%. In post-mortem studies, crypts were identified in up to 32% and were mostly localized in the anteroseptal region [25], matching our findings. Hence, the increased spatial resolution of 7.0T may lead to an improvement in the identification of small structures. Currently, the clinical advantage of myocardial crypt detection is unclear. There is first evidence, that the identification of ≥ 2 crypts had a 100% positive predictive value to identify carriers [8]. Another group could show that deep basal inferoseptal crypts are more common in patients with HCM with disease-causing mutations than in patients with genotype-negative HCM [22]. Assessment of family members is one of the most challenging and responsible tasks in a clinical setting. Detection of multiple crypts may add additional information.

The definition of crypts was based on access to the blood of the ventricles. All but one structure could be verified in perpendicular slices. In one patient (Fig 4f) we were not able to exclude, that this hyperintense region is related to a septal branch. But it is very unlikely, as the larger coronaries were not visible in the same intensity. A whole heart coverage would have been helpful to assess all anatomical details with 2D CINE imaging and should be used in future trials.

The detection of the inferobasal myocardial crypts is usually detectable in the two- chamber view, sometimes better in a modified one [8]. The assessment of additional crypts may depend on slice positioning. In the current trial we have focused on the most hypertrophied myocardial segments.

### Safety and technical aspects

All volunteers completed the CMR examination; no severe adverse events occurred matching pervious experiences [11]. The rate of minor subjective events was similar to recently published data [26]. Safety data about metallic implants are rare for 7.0T. At least dental wires, which many of the participants of the present study had, did not cause any problems. Other safety issues have to be addressed in the future to expand the use of 7.0T in cardiac patients. For example coronary stents are frequent in patients with coronary artery disease. The prevalence in US patients aged 20 and over is about 6.5% [27]. Currently the safety of CMR at ultrahigh fields (B_0_ ≥ 7.0 T) for patients with stents is under investigation. Recently, electro-magnetic field simulations and heating experiments at 7.0T demonstrated that radiofrequency (RF)-induced stent heating did not exceed limits given by the IEC guidelines for RF power deposition [28]. Another study scrutinized RF induced heating of coronary stents at 7.0T for a broad range of stent configurations [29].

Following the success of neuroradiology by detecting subtle focal lesions in the brain [30], CMR at ultrahigh fields may also allow us to detect histopathological structures of the heart. We were able to detect subtle myocardial structures at 7.0 T thanks to the spatial resolution which is superior to the capabilities of clinical field strengths. The differentiation of fibrotic areas in HCM might be helpful to identify prospectively patients with further development of systolic heart failure, but this was not a part of this pilot study. We anticipate, that CMR at 7.0T can also provide information on fibrotic tissue changes without any contrast application. First results have indicated, that at least assessment of changes in blood oxygenation could be possible [31].

## Conclusion

7.0T MRI is feasible in patients with HCM. High spatial resolution CINE imaging at 7.0T allows the identification of subtle morphological details in regions of extended hypertrophy and fibrosis. These structures were not detectable at clinical field strength and may allow new insights into the development of remodeling.

## Limitation

In this pilot trial the number of patients was limited; therefore a genotype/phenotype correlation is missing. This study has addressed the potential advantage of higher spatial resolution, whereas the challenges of acquisition time and systematic evaluation of magnetic field inhomogeneity have to be elucidated in next studies. RF power deposition was not a limiting factor in this setting, but could impact other CMR techniques such as fast spin-echo imaging.

In our setting we did not perform coverage of the entire LV by CINE-imaging due to examination time constraints. That may impact the assessment of further small structures. The use of high-resolution 3D cine application would help to overcome this limitation.

## Supporting Information

S1 File HCM patient with myocardial crypts at 7.0T. (MP4)
